# Devulcanization of Sulfur-Cured EPDM: A Critical Review of Selective Crosslink Scission, Its Measurement, and Continuous Processing

**DOI:** 10.3390/polym18202477

**Published:** 2026-10-10

**Authors:** Ki-Dong Nam, Yeon-Jee Cho, Ho-Dong Kim, Jeong-Hwan Yoon

**Affiliations:** 1 Exterior & Closure Materials Development Team, Hyundai Motor Company, Hwaseong-si 18280, Republic of Korea; 2 Sustainable Materials R & D Center, Korea Automotive Technology Institute, Cheonan-si 31214, Republic of Korea

**Keywords:** EPDM, devulcanization, crosslink scission, Horikx analysis, reactive extrusion, reclaiming, crosslink density, life cycle assessment, rubber recycling

## Abstract

Sulfur-cured ethylene–propylene–diene monomer (EPDM) sealing scrap cannot be melt-reprocessed; devulcanization recovers rubber only if it cuts crosslinks selectively. This review separates EPDM-direct findings from tire, natural rubber (NR) and styrene–butadiene-rubber transfers. Metrics have converged on swelling, crosslink density, sol fraction, Horikx selectivity analysis, and a rheological proxy, without a grading standard. Two EPDM studies favored milder temperatures; extrusion selectivity depended jointly on thermal profile and screw speed, and excessive severity can cause re-crosslinking. Thermomechanical and agent-assisted extrusion have run continuously on EPDM in more than one laboratory; one group’s agent-assisted devulcanizate met the tensile, compression set and hardness limits of a non-automotive seal specification, the last two only after blending with 25 wt% virgin rubber. In the only direct comparison, ethylidene-norbornene EPDM was much less reactive toward disulfides than NR, which its authors attribute to a denser, more monosulfidic network less prone to main-chain scission. A single EPDM process life cycle assessment (LCA) identifies energy as the main environmental lever. The review contributes maturity classes, scission pathways labeled proposed or demonstrated, and a minimum LCA reporting set. Closing five EPDM-direct gaps (grading standards, green-route data, in-line selectivity sensing, joint throughput–energy–quality reporting, feedstock sorting) would make continuous agent-assisted extrusion a specifiable route.

## 1. Introduction

Ethylene–propylene–diene monomer (EPDM) rubber is used in automotive weatherstrips, door seals, and anti-vibration components, applications that manufacturers list for its grades [1,2]. These uses rest on its resistance to ozone, oxygen, and elevated temperatures [3,4]. Its crosslinked network prevents melt reprocessing, so waste rubber sees limited reuse in new rubber products [4]. No EPDM-specific waste-arising inventory was found; quantities are reported for tires or for rubber in aggregate.

**Figure 1:**
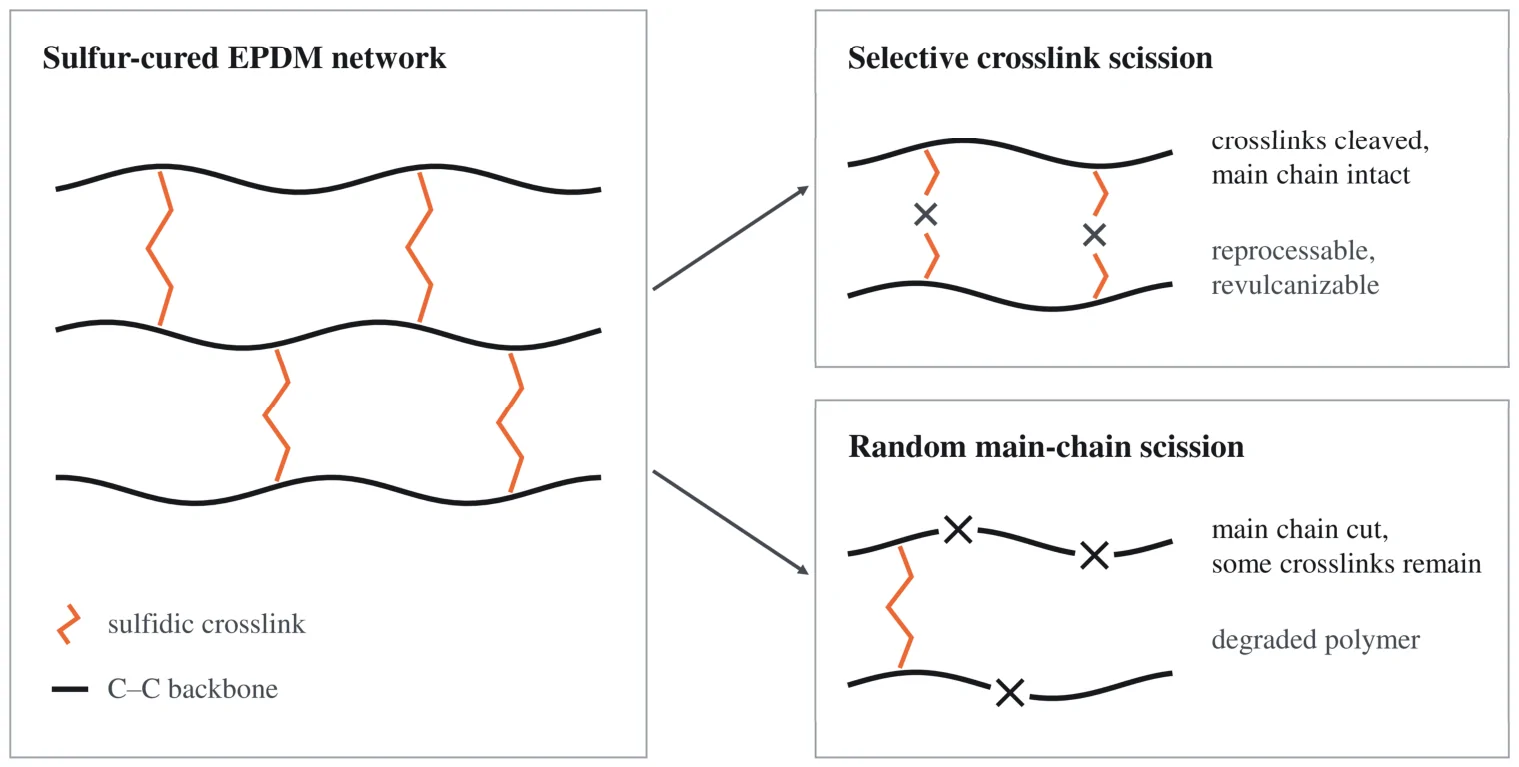
Selective crosslink scission and random main-chain scission in a sulfur-cured EPDM network (schematic). (**Left**) the vulcanized network; (**right**) two limiting outcomes: a reprocessable, revulcanizable devulcanizate with intact main chains, and a degraded polymer with cut main chains. Orange, sulfidic crosslinks; black, C–C backbone; crosses, cleaved bonds.

Devulcanization cleaves crosslinks selectively to restore a reprocessable, revulcanizable material while leaving the backbone as intact as possible (Figure 1). However, heat and shear do not confine scission to the weaker sulfidic crosslinks, and backbone bonds vastly outnumber them, so nearly every reported process cleaves some of both. The one complete network breakdown reported for EPDM, in an unfilled laboratory vulcanizate of sulfur-cured EPDM with 1,4-hexadiene as diene (HD-EPDM) heated with diphenyl disulfide at 265 °C, also involved main-chain scission [5]. Devulcanization is thus a trade-off between crosslink removal and polymer degradation that has to be measured. In the only direct comparison reviewed here, sulfur-cured EPDM with 5-ethylidene-2-norbornene as diene (ENB-EPDM), the common grade, was much less reactive toward disulfides than natural rubber (NR), which Verbruggen et al. [6] attribute to the higher crosslink density of the vulcanizates studied, a higher proportion of stable monosulfidic crosslinks and a lower propensity to main-chain scission. Whether EPDM’s low unsaturation, confined to the pendant diene, contributes is not established by the reviewed evidence, although the diene termonomer has been reported to change the outcome (Section 2.1).

Continuous agent-assisted EPDM devulcanization dates back at least to Toyota’s reactive twin-screw extrusion, followed by amine-fed twin-screw extrusion at 3–7 kg/h [7]. The technical and regulatory position has since changed. A disulfide route optimized in batch has run continuously at 270 g/h, and its devulcanizate met the compression set and hardness limits of a non-automotive seal specification only after blending with virgin rubber [8]. The same group’s batch work graded network breakdown on a common metric set [9]. The European Union (EU) end-of-life vehicle Regulation of July 2026 makes recycled content from post-consumer waste mandatory for plastics in new vehicle types; the target calculation excludes elastomers from tires and from thermosets other than polyurethane foams used for cushioning [10]. The Regulation’s text leaves open how vulcanized sealing rubber counts in that calculation.

Rubber recycling is well reviewed, from broad surveys in a largely tire-dominated frame [11,12,13,14,15,16,17] and process taxonomies [18,19,20,21] to in-depth maps of the tire branch [22,23,24,25,26,27,28,29,30,31]. Closest in subject are the EPDM reviews [4,32]. A recent broad survey closes by noting that “the types of rubber subjected to these methods are very limited. Focus has always been on waste tires and gloves, which indeed should also be diversified” [14]. EPDM differs from tire elastomers in cure chemistry and backbone unsaturation, so several tire-derived findings transfer only conditionally, and a synthesis that mixes the two implies an EPDM evidence base that does not exist.

This review takes up this diversification for sulfur-cured EPDM. Its primary objective is to establish how devulcanizate quality is graded, which network chemistry each process family delivers, and how far each has been carried toward continuous operation. The review differs from earlier ones in naming the substrate behind every finding, so that EPDM-direct results stay apart from transfers, in labeling each scission pathway as proposed or demonstrated, and in assigning each route a maturity class, noting where it rests on a single group or laboratory. Literature published up to 31 July 2026 was selected for its bearing on sulfur-cured EPDM; statements of absence are bounded by that literature, which is also likely to over-represent successful devulcanization. The review ends at a quality-graded devulcanizate, which is necessary but not sufficient for adoption; compounding and aging studies of EPDM devulcanizate have begun [33,34].

## 2. Feedstock and Pretreatment

Feedstock properties limit what any devulcanization process can achieve (Table 1). Crosslink chemistry determines whether selective scission is possible at all, and particle morphology sets how efficiently energy and any agent reach the network. Both are under-addressed for EPDM.

### 2.1. Crosslink Chemistry and Cure System Sorting

**Figure 2:**
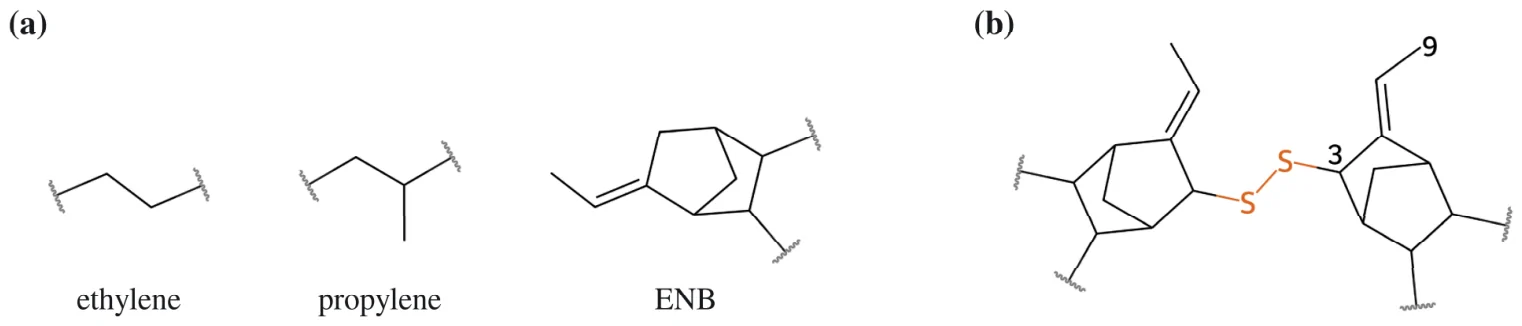
Structural units and sulfur crosslink of EPDM. (**a**) Ethylene, propylene, and ENB (5-ethylidene-2-norbornene) repeat units; the ethylidene double bond of ENB remains after copolymerization. (**b**) Two ENB units joined by a bisalkenyl sulfide crosslink S_x_ (x = 1, 2 or more; drawn for x = 2) at allylic carbon 3; carbon 9 is the other allylic position (numbering of [5]). Wavy bonds, continuation of the polymer chain.

EPDM has a saturated polymer backbone with pendant unsaturation in the diene monomer unit [3]. In the most common grades, the diene is ENB, whose ethylidene double bond remains after copolymerization and is used for crosslinking (Figure 2a) [5]. Sulfur vulcanization links the chains through mono- and oligosulfidic crosslinks, bisalkenyl sulfides attached at the allylic position that leave the double bond itself unconsumed (Figure 2b) [5]. Sulfidic crosslinks (S–S, C–S) are weaker than the C–C backbone and can be preferentially cleaved, whereas peroxide-derived C–C crosslinks generally cannot [3,35,36]. The exception found is peroxide-cured HD-EPDM, which devulcanized by crosslink scission, while peroxide-cured ENB-EPDM broke down mainly by random scission [5]. Therefore, a scrap stream’s devulcanizability is set largely by its cure chemistry before any process is chosen. Peroxide-cured ENB-EPDM, valued for the thermal stability of its C–C network [3], is a poor feedstock and, mixed into a sulfur-cured stream, would likely dilute achievable selectivity.

In a heated or sheared network, selective S–S and C–S scission competes with random main-chain scission and, in some systems, with re-crosslinking: in EPDM, by amine-promoted sulfur crosslinking above about 240 °C in one compound [37], by residual diene unsaturation in EPDM with dicyclopentadiene as diene (DCPD-EPDM) [5] and, at the higher of two peroxide doses, by a proposed macroradical recombination [38]. The bond energy values quoted for C–C, C–S and S–S bonds differ between sources, though their order does not [14,39,40]. Citing earlier work, one EPDM study argues that, because those energies are of similar magnitude, the larger spread in the bonds’ elastic constants lets shear, unlike heat alone, break sulfur crosslinks preferentially [41]. Agent routes add their own pathways, two demonstrated on EPDM in single studies (Section 4).

Crosslink density and rank matter beyond the cure class. In thermal treatment with hexadecylamine, termed reclaiming by the source, efficiency depended strongly on both crosslink density and its distribution. Contrary to the expectation that poly- and disulfidic networks devulcanize more readily, the efficiently cured, largely monosulfidic compound reached low Mooney viscosity under thermal treatment alone, whereas the conventionally cured compound required the amine. Horikx analysis in the same study nonetheless assigned, qualitatively, the breakdown of the efficiently cured compound mainly to main-chain scission and that of the conventionally cured compound mainly to crosslink scission [37], so the lower Mooney viscosity did not signal more selective devulcanization. The cure severity and accelerator system, which set crosslink density and rank together, are therefore feedstock variables in their own right, although sulfide interchange may rapidly convert the original rank at devulcanization temperatures [5].

The diene termonomer also affects the outcome. In unfilled laboratory vulcanizates heated at 200 or 265 °C, sulfur-cured ENB-EPDM devulcanized mainly by crosslink scission. Sulfur-cured HD-EPDM broke down by scission of both crosslinks and main chains, which the authors consider more likely to be main-chain scission next to crosslink sites (Horikx’s directed scission; Section 3.2). Sulfur-cured DCPD-EPDM could not be devulcanized but crosslinked further, which the authors attribute to residual DCPD unsaturation. The three vulcanizates, cured with one conventional sulfur recipe, also differed in crosslink density and sulfur rank, which the authors could not equalize [5]. Devulcanization chemicals raised the yield only where thermal devulcanization already occurred [5].

For ENB-EPDM, it has been proposed that a sulfur bridge attached at either allylic position, C9 or C3 (Figure 2b), is folded toward the other by the rigid norbornane, favoring crosslink scission through a cyclic sulfide, a route not open to DCPD-EPDM [5]. The ENB-EPDM vulcanizates in the comparison of Section 1 were also less reactive than NR under identical thermochemical conditions [6], so ENB-EPDM is likely to need more severe or better-targeted conditions than NR-derived rules of thumb suggest.

Cure system sorting is therefore a prerequisite, yet identifying crosslink type, let alone rank distribution, in heterogeneous post-consumer scrap is difficult. For tires, no technology separates rubber by polymer or formula, and sorting goes by dimension alone [42]. No protocol for sorting EPDM scrap by crosslink type appears in the reviewed material, although thiol-amine chemical probes have been used to determine crosslink rank in EPDM [6,37].

### 2.2. Size Reduction and Particle Morphology

In the tire rubber literature, the grinding route (cryogenic vs. water-jet) sets particle size and specific surface area (SSA), which influence the devulcanized product, and devulcanization in turn promotes further size reduction [43,44]. Industrial cryogenic and ambient grinding of tires have been compared for capacity, energy requirement, and particle size distribution, and tire crumb has been characterized [45,46].

For EPDM crumb from used tires, particle size was an important process parameter, and particle size distribution correlated strongly with the energy distribution in the process, consistent with a diffusion-controlled step tied to specific surface area [47]. The same dependence is documented for styrene–butadiene rubber (SBR) regeneration [48]. A diffusion-limited surface step implies that a coarse particle leaves an unreacted core; in reclaiming of two EPDM compounds, results indicated peeling of outer particle layers, mainly in one compound, with the cores largely untreated, and in the other a finer initial granulate gave slightly better properties [37]. However, in the SBR study, crosslink density reduction was favored by larger particles, so the size–outcome direction is not settled. The size–outcome relationship for a given EPDM sealing scrap formulation is unmapped.

Two gaps remain in EPDM feedstock preparation: provenance, and pretreatment evidence transferred from tires. The EPDM-direct results rest largely on post-industrial seal scrap [8,39] or laboratory-cured compounds [41], for which cure system sorting does not arise, whereas a circular stream would include post-consumer parts of mixed formulation, filler, and cure chemistry, and sorting and contamination control for this harder feedstock are unaddressed. Whether EPDM’s distinct network and filler respond to grinding like tire rubber [43,44,49] is untested. The processes reviewed below therefore presume a well-characterized, correctly sorted, appropriately comminuted input that the EPDM recycling chain does not yet reliably deliver.

**Table 1:** Feedstock and pretreatment attributes governing EPDM devulcanizability, with the state of EPDM-specific evidence. Substrate labels as in Table A1 (EPDM(t), EPDM crumb of tire origin; tr, tire rubber transfer); DBD, dibenzamido-diphenyl-disulfide.

Attribute	Effect on Devulcanization	Direction/Evidence (Substrate)	EPDM-Specific Status	Refs.
Crosslink type (sulfur vs. peroxide)	Sets whether selective scission is possible	Sulfidic S–S and C–S cleavable; peroxide C–C not selectively cleavable, except in HD-EPDM (EPDM, NR)	Established (EPDM + transfer)	[3,5,35,36]
Crosslink rank distribution (mono-, di-, poly-)	Higher-rank bridges generally break preferentially; low-concentration DBD reportedly cleaves monosulfidic bridges first (stress relaxation inference)	Efficiency depends on crosslink density and its distribution (EPDM); original rank possibly irrelevant at devulcanization temperatures (EPDM)	EPDM-direct, conflicting	[5,37,50]
Polymer and network reactivity	Sets the severity needed for a given scission	ENB-EPDM much less reactive to disulfides than NR in the only direct comparison, attributed to crosslink density, monosulfidic share, and lower main-chain scission (EPDM, NR)	EPDM-direct, one comparison	[6]
Particle size/specific surface area	Governs reagent and shear access; direction unsettled	Size an important parameter; size distribution tracks energy distribution (EPDM(t), SBR); outer-layer peeling, finer granulate slightly better (EPDM)	Mostly tire origin; one EPDM-direct study	[37,47,48]
Grinding route/morphology	Sets particle size distribution and SSA	Cryogenic and water-jet grinding differ; morphology influences outcome; devulcanization aids further comminution (tr)	Transfer only	[43,44]
Feedstock provenance	Post-industrial (known) vs. post-consumer (mixed)	Several EPDM-direct studies use post-industrial seal scrap or a laboratory-cured compound; post-consumer data limited to mixed automotive powder (EPDM) and used-tire EPDM crumb (EPDM(t))	Gap: no sorting protocol	[8,39,40,41,47,51]

## 3. Quantifying Devulcanization Quality

The difficulty is to achieve selective network breakdown and to demonstrate that it was selective. Because heat and shear break sulfidic crosslinks preferentially, but backbone bonds far outnumber them, the processes reviewed here cleave a mixture of sulfidic crosslinks and main-chain C–C bonds. Therefore, devulcanizate quality is a position on a trade-off surface between crosslink removal and polymer degradation [35,39]. The EPDM literature has converged on a metric set of equilibrium-swelling crosslink density, sol/gel fraction, Horikx (Charlesby–Horikx) selectivity analysis, and rheological descriptors. The set grades that trade-off only in combination and was not built for devulcanization. Its pillars are 1940s–1950s solution and network swelling thermodynamics [52,53,54] and network degradation analysis. The latter runs from Charlesby’s solubility treatment [55] through Horikx’s scission analysis [56] to the Charlesby–Pinner relation [57]. The set’s caveats, among them sensitivity to the assumed pretreatment state, a neglected third scission mode, and the entanglement contribution to swelling-derived densities, are inherited from those derivations.

### 3.1. Crosslink Density and Sol/Gel Fraction

The primary measurand is the crosslink density *ν* from equilibrium swelling via Flory–Rehner analysis [35,39,52,53,54,58]. The analysis balances network elasticity against polymer–solvent mixing through the interaction parameter *χ*. Quoted *ν* values are therefore conditional on the *χ* assumed for the rubber–solvent pair [56], and the measured quantity is an effective chain density including trapped entanglements (Table 2). Its decrease relative to the starting vulcanizate is the headline indicator, and percent devulcanization follows from the *ν* ratio, standardized for crumb rubber in ASTM D6814 [59] and applied to EPDM crumb in modified form [47].

A swelling-based *ν* is meaningful only once extractable non-network ingredients (oil, lubricants, processing aids) are separated from the polymeric sol. This correction is formalized in the EPDM literature [35] and was already present in the original swelling-plus-scission measurements [56], hence intrinsic to the method. Reinforcing filler requires a second correction. In carbon-black-filled EPDM, the apparent *ν* of the virgin vulcanizate rises with filler loading at nearly constant gel fraction, even after Kraus correction [60]. Some EPDM studies correct swelling data for filler with the Kraus relation [37,38]; without a declared correction, extents quoted for filled and unfilled compounds are not directly comparable.

The sol fraction is the complementary, cheapest indicator, and it is read by direction. Low sol with a large *ν* decrease indicates selective crosslink scission, whereas high sol at a moderate *ν* decrease signals main-chain degradation; crosslink scission alone releases little sol until most crosslinks are broken. Its formal basis is the sol fraction–crosslinking index relation of the statistical treatment of randomly crosslinked polymers [55,56]. This relation lets a single extraction carry network connectivity information without empirical calibration.

### 3.2. Horikx Analysis: Scope and Limitations

**Figure 3:**
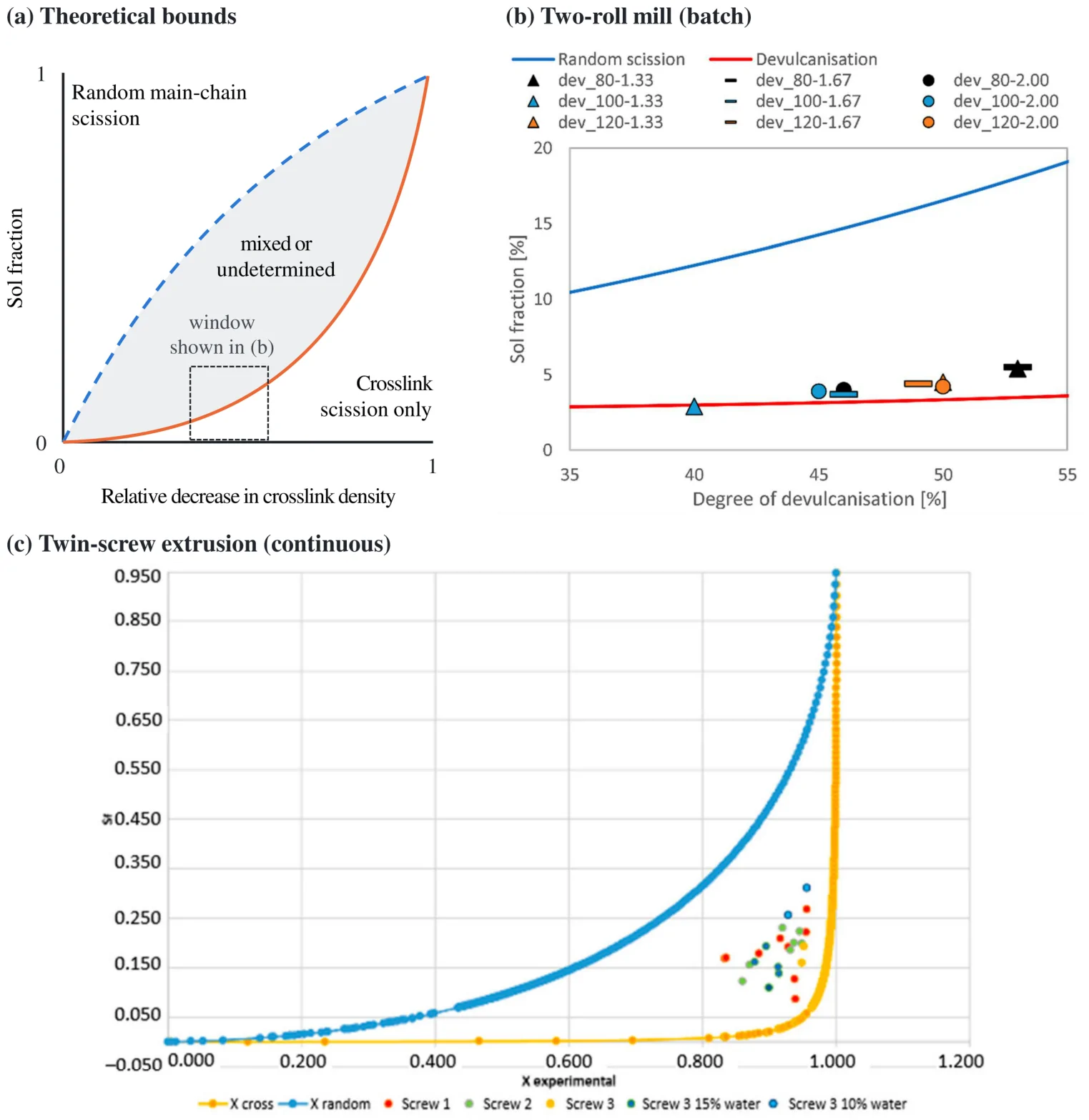
Horikx analysis of EPDM devulcanization. (**a**) Schematic curves, not computed, for random main-chain scission (dashed) and crosslink scission only (solid) [56]; shaded, mixed, or undetermined; dashed box, approximate window of (**b**), curve heights not to scale. (**b**) Two-roll mill; codes, mill temperature (°C)–friction ratio; “Devulcanisation”, crosslink scission curve; “degree of devulcanisation”, relative decrease in crosslink density. Adapted from Pirityi and Pölöskei [35], https://doi.org/10.3390/polym13071116, © 2021 by the authors, licensee MDPI, Basel, Switzerland, CC BY 4.0 (https://creativecommons.org/licenses/by/4.0/); cropped from the source Figure 2, content unaltered. (**c**) Twin-screw extrusion; X experimental, relative decrease in crosslink density; Sf, sol fraction; X cross, X random, crosslink scission and random scission curves; points, screws 1, 2, and 3, and screw 3 with 15% or 10% water injection. Adapted from Brunella et al. [39], https://doi.org/10.3390/polym14224853, © 2022 by the authors, licensee MDPI, Basel, Switzerland, CC BY 4.0 (https://creativecommons.org/licenses/by/4.0/); cropped from the source Figure 8, content unaltered.

The Horikx plot (Figure 3) is the selectivity analysis used most often in the EPDM work reviewed here [35,36,39,58]. It plots sol fraction against the relative decrease in crosslink density, bracketed by the theoretical curves for purely random main-chain scission and purely crosslink scission. The original derivation [56] defines both the scope and the limits of that interpretation. It distinguishes three idealized scission modes, namely random-chain scission, severance of the crosslinks, and directed scission of monomer units adjacent to a crosslink. Only the first two admit closed-form treatment. Directed scission receives a relation its author states is “very approximate” and on which conclusive evidence cannot be based [56]. The modern diagram plots the two closed-form bounds, and a trajectory between them is conventionally interpreted as a mixture of random and crosslink scission. A directed scission contribution would also fall there, so an intermediate position constrains the mechanism without resolving it. Therefore, the position in the Horikx plot is a selectivity indicator whose mechanistic interpretation requires independent chemical evidence. A quantitative treatment of directed scission remains an open item in the theory.

Measurement practice is governed by an asymmetry between the two closed-form relations. The random scission curve is insensitive to the initial crosslinking index, whereas the crosslink severance curve, the curve that grades selectivity, is “very sensitive” to the pre-scission network state [56]. The practical rule is therefore to take the crosslinking index from the sol fraction before devulcanization. Deriving it from swelling-based chain density, which responds to entanglements as well as chemical crosslinks, inflates it [36]; the index in Figure 3c was swelling-derived [39].

The original curves were checked only against thermo-oxidative degradation of sulfur/sulfenamide-cured natural rubber [56], so the transfer of the analysis to devulcanization is an inference. Purpose-built validations narrow that gap. Partially cured samples, standing in for devulcanized ones, reproduced the crosslink scission curve across 0–100% nominal devulcanization for sulfur- and peroxide-cured EPDM and sulfur-cured NR with and without carbon black, all mineral-filled [58]. The merged Charlesby–Horikx model was also confirmed with deliberately selective chemistries. Thiol-amine reagents, which break sulfur crosslinks exclusively and cause no main-chain scission in NR or EPDM, were compared against peroxide-cured NR, which breaks down by random scission. The data fitted the model “surprisingly well”, but EPDM was tested only under crosslink scission, with *χ*-dependent deviation for ENB-EPDM at large decreases [36].

Filler perturbs placement in the plot. In ultrasonically devulcanized EPDM, carbon black moved gel–crosslink density data toward the main-chain-scission limit of a network statistics model [60]. A selectivity claim for filled industrial scrap is therefore weaker evidence than for a gum compound, and grading should state the filler context. Selectivity also extends to crosslink rank. Solid-state shear milling of ground tire rubber broke poly- and disulfidic crosslinks preferentially while leaving monosulfidic concentration largely unchanged, and the residual population makes the devulcanizate chemically dissimilar from virgin rubber and affects revulcanization [61]. Crosslink density *ν* and Horikx analysis, aggregating all crosslinks into one number, are blind to rank. Horikx analysis also assumes homogeneous breakdown throughout the particles; where outer layers are peeled while the cores stay largely untreated, the points shift and cannot be read directly against the theoretical curves [37].

**Table 2:** Devulcanization quality metrics for EPDM: measurand, method, and key caveat. *ν*, crosslink density; *χ*, polymer–solvent interaction parameter.

Metric	Measurand	Method Basis	Key Caveat	Refs.
Crosslink density decrease Δ*ν*; % devulcanization	*ν* before/after	Equilibrium swelling, Flory–Rehner; ASTM D6814, commonly adapted	Conditional on assumed *χ*; responds to entanglements; oil/aid extraction correction; filler raises apparent *ν*; reference state must be declared; % can be negative in batch mixing	[35,39,47,52,53,54,56,58,59]
Sol/gel fraction	Extractable polymer	Soxhlet/solvent extraction; sol–crosslinking index relation	Non-network ingredients inflate sol unless corrected	[35,55,56,57,60]
Selectivity	(sol, Δ*ν*) trajectory	Horikx/Charlesby–Horikx plot	Two curves, three scission modes, directed scission unresolved; crosslinking index *c_i_* from pre-devulcanization sol, not swelling; filler shifts placement; assumes homogeneous breakdown	[35,36,37,56,58]
Processability proxy	Viscosity, modulus, torque	Mooney, MDR; in-line use not yet reported	Correlates with, but does not certify, selectivity; set point ≠ material temperature	[9,62]

### 3.3. Rheological Proxies and Grading Standards

Swelling-based metrics require extracted samples in the laboratory, and none of the studies reviewed here ran them in line. The literature complements them with rheological descriptors (Mooney viscosity, and the storage modulus and the re-compounded material’s torque in a moving die rheometer (MDR)), which track devulcanization extent and processability [9,62]. Gray-box soft sensors predict melt viscosity in polymer extrusion generally [63], but none reviewed here targets devulcanizate quality. Mapping process parameters onto sol–gel analyses and revulcanization properties in one design space [9] is the pattern a grading standard needs. It pairs a laboratory anchor (*ν*, sol, position in the Horikx plot) with a continuously monitorable proxy.

Where process conditions are used to index quality, the material temperature differs from the nominal set point. In a twin-screw devulcanization of post-production NR waste at 5 kg/h, on a semi-industrial extruder (the source’s term), barrel temperatures set to 80 and 100 °C corresponded to material temperatures of approximately 155 and 145 °C through self-heating [62]. Raising the input temperature in that study induced degradation and did not increase devulcanization. Allowing for the substrate difference, two implications carry to EPDM: the barrel set point does not fix the material state, and a hotter barrel does not necessarily give more, or more selective, devulcanization.

Although the metric set has stabilized (Table 2), no grading standard exists. Studies differ in solvent, extraction protocol, filler treatment, and reference state, hampering cross-study comparison, including the comparisons collected in Table A1. The headline “degree of devulcanization” is not robust across equipment. For EPDM crumb from used tires, twin-screw extrusion gave degrees of regeneration of 80–87% with shear intensity dominant [47]. In the same study, an internal batch mixer produced both positive and negative degrees, with treated particles registering a higher apparent crosslink density than untreated ones. Because the sign changed with rotor speed alone, the degree cannot yet serve as a grade. Reported degrees are also conditional on comminution history as well as severity (Table 1), and neither is routinely declared. A high nominal degree does not by itself establish selectivity: the NR twin-screw study reported approximately 90% reclaiming, largely independently of barrel temperature [62].

No widely adopted grade classes (e.g., selectivity bands in the Horikx plot) exist for devulcanized EPDM as an industrial feedstock. The standards that would anchor grading, liquid exposure/swelling testing [64] (ISO 1817) and crumb rubber percent devulcanization [59] (ASTM D6814), are cited unevenly, applied in adapted form [47], and are not EPDM-specific. Emission-based attributes are also absent from the EPDM metric set. The wider literature, by contrast, proposes standardized volatile organic compound (VOC) protocols, with benzothiazole, limonene, and BTEX (benzene, toluene, ethylbenzene, xylenes) markers, as a product quality tool and notes that recyclates can, in some cases, exceed regulatory emission limits by large margins [65]. A framework combining the four converged metrics with declared filler context, comminution history, actual (not set point) material temperature, and measurement protocol would make devulcanizate quality portable between laboratories and specifiable in commercial terms.

## 4. Process Families

**Figure 4:**
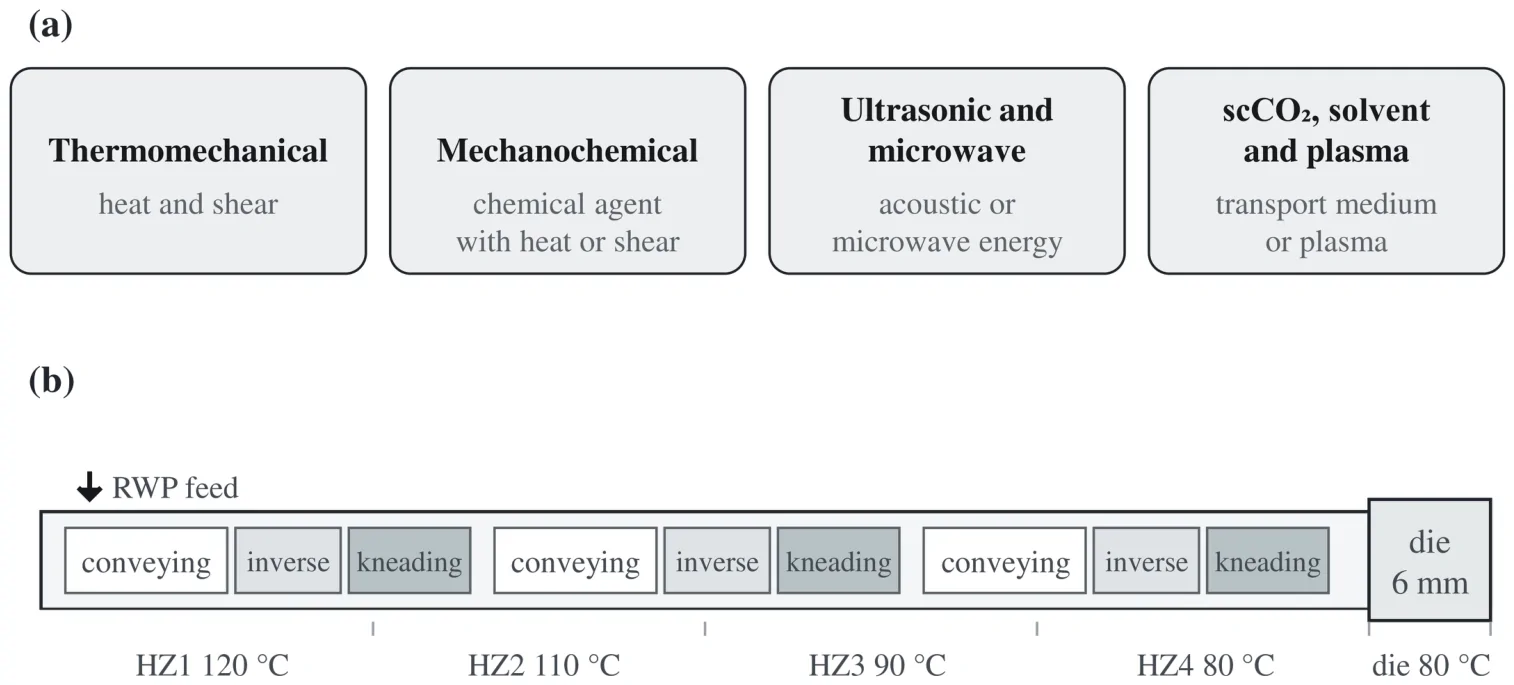
Process families and a continuous twin-screw line. (**a**) Devulcanization families and their means of network scission; scCO_2_, supercritical CO_2_. (**b**) Screw configuration and heating zone (HZ) temperatures reported by Gschwind and Jordan [8], not to scale; rubber waste particle (RWP) feed, three conveying–inverse–kneading sections, zones at 120, 110, 90, and 80 °C, and an 80 °C, 6 mm die.

Devulcanization breaks sulfidic crosslinks and ideally leaves the hydrocarbon backbone intact, whereas in reclaiming main-chain scission occurs along with crosslink scission [37]. However, the two terms are often used interchangeably [21]. In vulcanized rubber, devulcanization and degradation are interrelated and often occur simultaneously, so crosslink cleavage and main-chain scission cannot be targeted independently during processing [66]. In this review, degradation denotes the unintended main-chain (C–C) scission that accompanies devulcanization and prevents recovery of the original macromolecules [18], whereas depolymerization denotes backbone cleavage pursued as the aim, as in olefin metathesis or selective oxidative cleavage to oligomers or low-molecular-weight polymers [19,66]; pyrolysis goes further, to oil, gas, and char [20]. Usage also varies: one review calls chain scission in reclaiming depolymerization [16], and the ASTM definition cited by Macsiniuc et al. [47] counts depolymerization as part of devulcanization. Table 3 summarizes these process classes, including biological devulcanization, and Figure 4a shows the devulcanization families reviewed below, with their means of network scission. Chemical devulcanization is very often combined with other techniques, a combination held necessary to limit main-chain degradation [18].

For disulfide-type routes, NR and tire results may overstate the ease of devulcanizing EPDM. In the only direct NR–EPDM thermochemical comparison with disulfides, NR sulfur vulcanizates were completely plasticized at 200 °C, whereas ENB-EPDM was much less reactive under the same conditions [6]. Unfilled sulfur-cured HD-EPDM, a less common grade, by contrast, devulcanized almost completely at 200 °C with diphenyl disulfide [5]. The direction of this bias is route-dependent. For supercritical CO_2_ (scCO_2_), no EPDM-direct performance evidence exists (Table 3), so a tire-derived process window may overstate or understate EPDM performance. Whether EPDM’s lower reactivity, which also slows main-chain scission, widens the selectivity window is not established by the EPDM data.

### 4.1. Thermomechanical Processing

Thermomechanical devulcanizate quality is confined to a process window bounded by main-chain scission at excess severity and by a network too crosslinked to reprocess at insufficient severity. Heat and shear alone can be applied on equipment compounders already own, which is the route’s industrial appeal [35,41]. In a two-roll-mill study, Horikx analysis rated the devulcanizate made at 80 °C and a friction ratio of 1.33 best, with a ~53% crosslink density decrease at 5.4 wt% sol [35] (Figure 3b; Table A1). The temperature response was not monotonic, 100 °C being the least effective. On one sulfur-cured profile-grade EPDM and one machine set, batch processing (mill plus internal mixer) reached a 75% crosslink density decrease against 72.5% for twin-screw extrusion, which caused slightly more chain degradation [41]. Revulcanizates with up to 50 wt% of the batch devulcanizate retained the original tensile strength with a slight modulus increase. In continuous twin-screw processing, the thermal profile, screw speed, and screw configuration shift the sol fraction and the share of random scissions [39].

The continuous extrusion study cites a model from Toyota’s continuous reactive extrusion of sulfur-cured EPDM, in which heat converts poly- and disulfidic crosslinks into monosulfidic ones and shear then breaks them [32,39]. The model is consistent with the monosulfidic enrichment measured in thermally treated EPDM [6,37] (Table A1). The batch-versus-continuous study names radical formation, rearrangement, substitution, and oxidation as the generally accepted main pathways, which occur simultaneously, cannot be isolated, and are favored differently by processing [41]. The study adds that they are not fully understood, so they remain proposed. In the same source, air exposure above 240 °C mainly drives oxidative degradation, and exposure above 280 °C can trigger random-chain scission through radical formation [41], which makes atmosphere a process variable. The NR–EPDM comparison, for example, devulcanized EPDM in a closed mold under nitrogen [6].

Re-crosslinking is a third outcome of excess severity. In an efficiently cured, largely monosulfidic EPDM, hexadecylamine acted above about 240 °C as a crosslinking promoter and not as a devulcanization aid, which the source relates to the known enhancing effect of amines on sulfur crosslinking [37]. In SBR devulcanized with diphenyl disulfide, the sol fraction rose with temperature but fell again at 260 °C, while crosslink density rose [73]. In EPDM roofing waste, Colom et al. [38] proposed that macroradicals formed in scission recombine, a “cage effect”, to explain why 4 phr of peroxide reduced crosslinking less than 2 phr did.

Continuous EPDM-direct twin-screw operation is reported by two laboratories [39,41] (Table A1), and the process of the first has run on a 120 kg/h production line without reported devulcanizate quality [74] (Table A2). On tire rubber, continuous processing studies [75,76,77,78,79,80,81] report screw speed, feed rate, screw configuration, and barrel temperature as variables that shift the outcome.

### 4.2. Mechanochemical (Agent-Assisted) Processing

**Figure 5:**
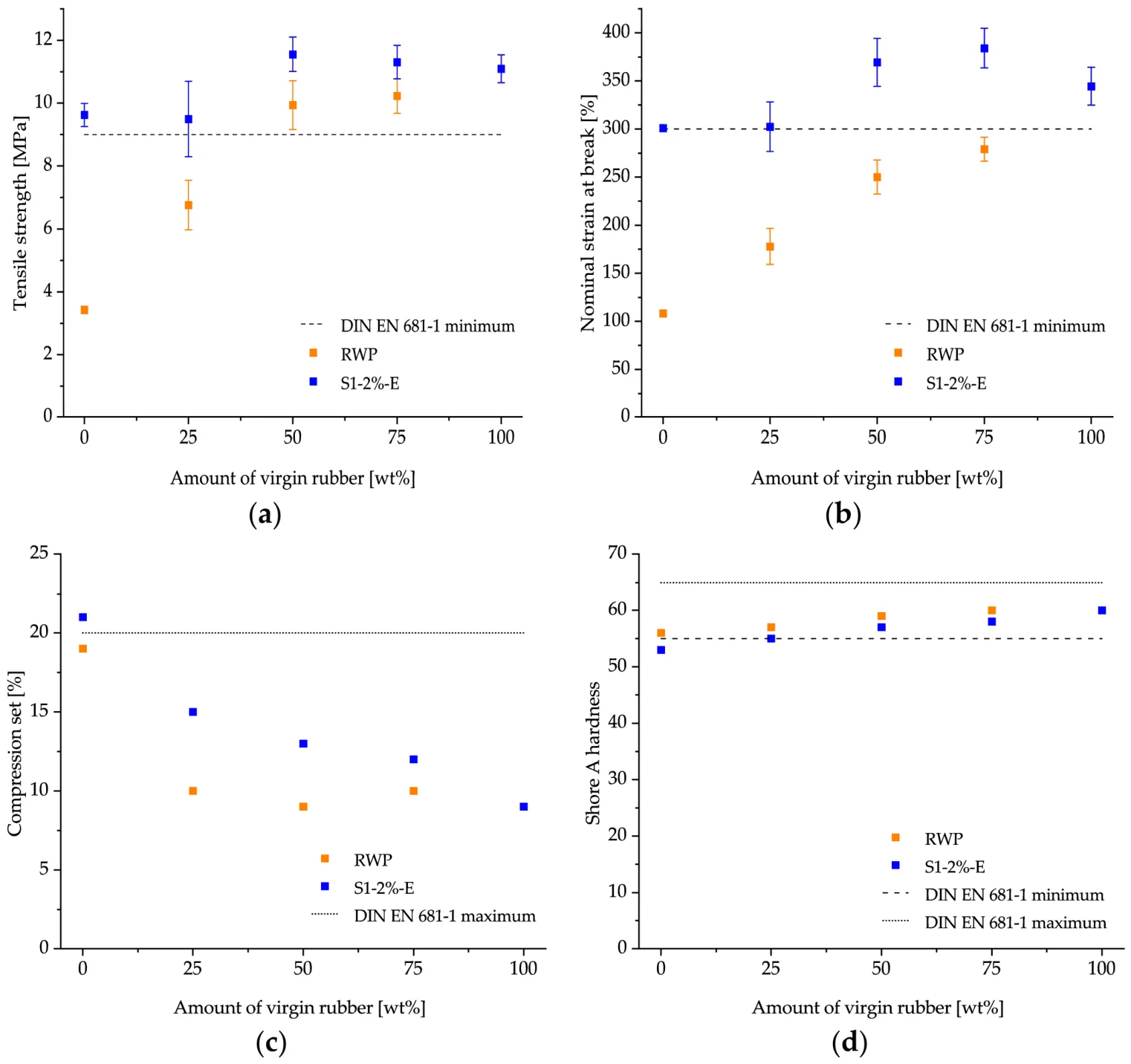
Devulcanized EPDM and untreated rubber waste across blend ratios. Tensile strength (**a**), nominal strain at break (**b**), compression set (**c**) and Shore A hardness (**d**) of blends of virgin rubber (VR) with untreated rubber waste particles (RWP, orange) and with the extruded devulcanizate S1-2%-E (blue), 0–100 wt% VR; the 100 wt% point is virgin rubber alone. Dashed and dotted lines: DIN EN 681-1 requirement levels as reported by Gschwind and Jordan [8], minimum or maximum as labeled. Adapted from Gschwind and Jordan [8], https://doi.org/10.3390/recycling8010008, © 2023 by the authors, licensee MDPI, Basel, Switzerland, CC BY 4.0 (https://creativecommons.org/licenses/by/4.0/); cropped from the source Figure 10 to panels (**a**–**d**), content unaltered.

On EPDM, the gain an agent brings at a given severity depends on the agent and the compound. The most complete EPDM dataset, from one group, concerns dibenzamido-diphenyl-disulfide (DBD). In a shear-free heated press, devulcanization of ground EPDM waste depended strongly on DBD concentration and temperature [50]. In a comparison of press, internal mixer and co-rotating twin-screw extruder, 2 wt% DBD in the extruder with a falling temperature profile performed best after upscaling from 40 g batches to 270 g/h continuous operation [8] (Figure 4b; Table A1 and Table A2). The recyclate met the tensile strength requirement of DIN EN 681-1:2006 [82], a non-automotive specification for vulcanized rubber pipe-joint seals in water and drainage service. Elongation was inferred from nominal strain, whereas the standard prescribes an extensometer method. Compression set and hardness met the requirements only after blending with 25 wt% virgin rubber (VR), the blend reaching 55 Shore A [8] (Figure 5). The study also discusses DBD as a lower-odor alternative to diphenyl disulfide.

The follow-up study maps process parameters against sol–gel and revulcanization properties [9]. In the most recent study, tensile strength and strain at break rose with network breakdown as inhomogeneity fell, apart from a strain-at-break drop between 53% and 64% devulcanization that the authors attribute to residual DBD [83] (Table A1). A liquid polymer activator improved both properties at low network breakdown, worsened them at moderate breakdown, and had little effect at high breakdown.

With 2-mercaptobenzothiazole disulfide (MBTS) and process oils, a semi-industrial twin-screw extruder (nameplate 100–250 kg/h) devulcanized waste automotive EPDM powder to about 92% at 13–18 wt% sol [51] (Table A1). In an industrial steam autoclave, run batchwise on oil-soaked automotive EPDM powder, MBTS reached 94% devulcanization against 76% with TMTD at the same oil content and the higher steam pressure [40] (Table A1).

In internal mixer treatment of a conventionally cured (primarily poly- and disulfidic) EPDM with the reclaiming agent hexadecylamine at 225–275 °C, the crosslink density decrease came mainly from the treatment itself, the amine adding little [37] (Table A1). However, treatment without the amine was insufficient, and a noticeable network breakdown with measurable Mooney viscosity required heat and amine together. The source cites earlier work finding α-hydrogen-bearing aliphatic amines more effective than the diaryl disulfides used for NR. These disulfides devulcanize EPDM only to some extent and leave a damaged devulcanizate that is insufficiently active in revulcanization.

Most agent pathways in these sources are stated as hypotheses; two are demonstrated in single EPDM studies, by chemical-probe crosslink analysis for an amine and by product analysis for diphenyl disulfide. For DBD, the sol–gel study offers two hypotheses [9]. Either DBD attacks the double bond adjacent to the crosslinking site, cleaving the network and re-forming an active crosslinking site at the chain end, or temperature or shear cleaves polymer and DBD alike, and DBD then acts as a radical scavenger. Grinding, shearing, and DBD cannot be told apart as causes of main-chain scission. For MBTS, the proposed scheme has shear or steam break chains and crosslinks while MBTS splits into resonance-stabilized radicals [40,51]. These combine with the chain radicals to leave accelerator-terminated polysulfidic pendant groups ready for revulcanization, and process oil is assigned a radical acceptor role against gel formation.

For the amine, poly- and disulfidic crosslinks broke preferentially, some becoming monosulfidic, and the amine’s distinctive contribution was to break thermally stable monosulfidic crosslinks, though fewer than heat formed [37]. An ionic pathway for hexadecylamine has been proposed only for tire rubber under microwave irradiation [84].

The one EPDM-direct product analysis of an agent route, for diphenyl disulfide at 200–275 °C, provides demonstrated evidence [6]. After devulcanization at 275 °C, gas chromatography–mass spectrometry (GC–MS) of the acetone extract found EPDM degradation products, benzenethiol and methylthiobenzene, attributed to scavenging reactions, and diphenyl monosulfide, taken as evidence that the disulfide transfers sulfur. Heating at 275 °C without disulfide left 91% of the surviving crosslinks monosulfidic. For both NR and EPDM, the authors concluded that “selective scission of sulfur crosslinks does not seem possible with this devulcanization system” [6].

**Figure 6:**
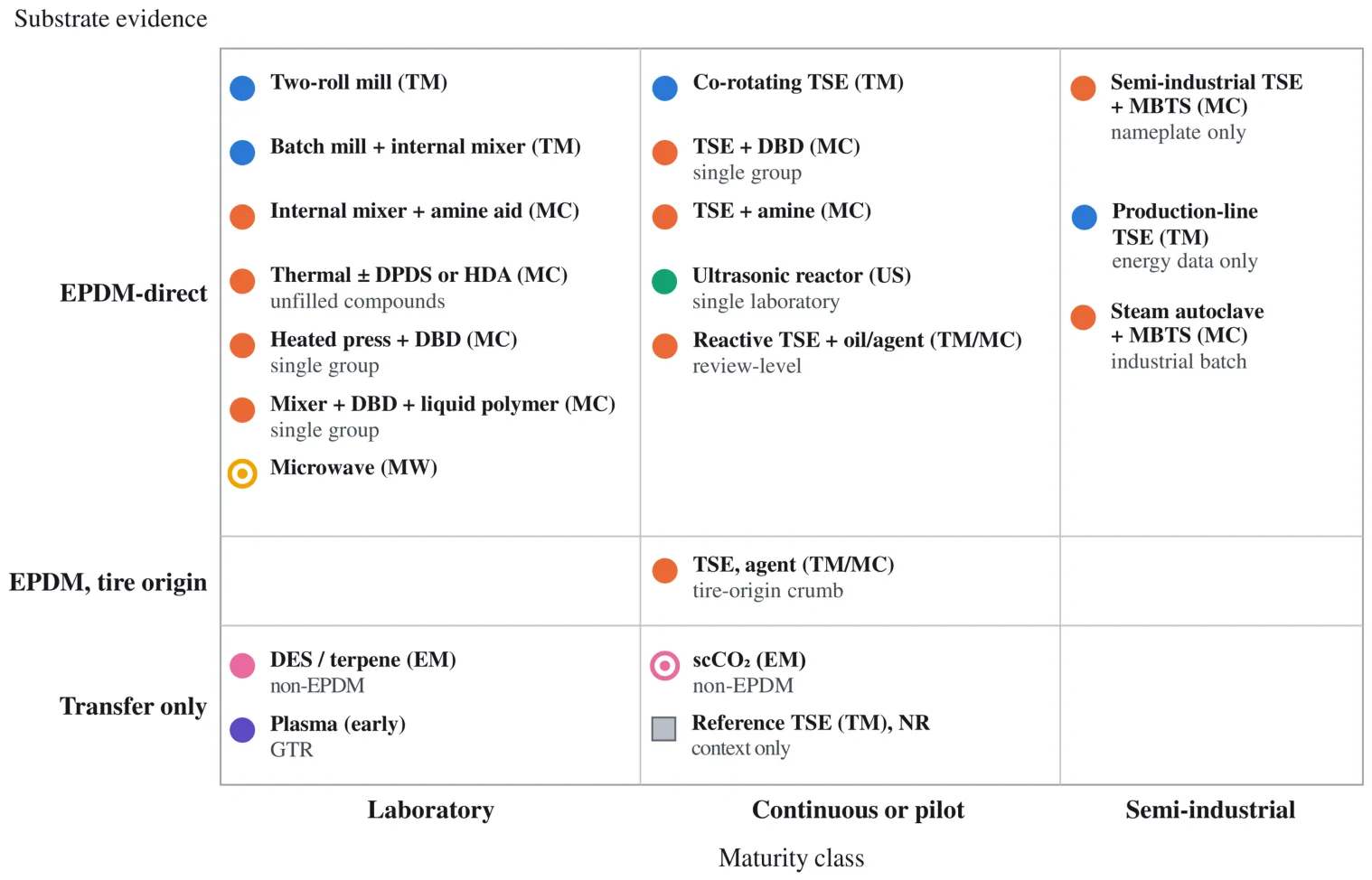
Maturity map of EPDM devulcanization routes. Classes as defined in Section 4.5. Markers carry the route labels of the Appendix A, Table A1 studies that set their position, whose references are listed there; marker color, route code (TM, thermomechanical; MC, mechanochemical; US, ultrasonic; MW, microwave; EM, emerging/solvent-assisted; violet, plasma; TM/MC rows in the MC color); text after a dash, the limit on that evidence. Ringed markers, several studies but no common grading (MW) or pilot equipment on a non-EPDM substrate (scCO_2_); gray square, context only.

The DBD evidence has not been independently replicated (Figure 6; Table A1). A silane-based tetrasulfide that cleaves NR networks and pre-functionalizes the devulcanizate for silica reinforcement transfers to EPDM only as a bifunctional design concept [85]. The largely non-EPDM agent literature often treats the route as one stage of a longer recycling sequence [86,87,88,89].

### 4.3. Ultrasonic and Microwave Devulcanization

Ultrasonic devulcanization has the most developed process modeling of the specialized equipment families, but the least resolved chemistry. Ultrasonic reactors deliver scission energy acoustically and supply the most detailed filler–devulcanization data in the reviewed literature [60]. In a continuous grooved-barrel reactor, the degree of devulcanization rose with ultrasonic power and amplitude, and normalized gel fraction and crosslink density fell with amplitude. Carbon black lowered the ultrasonic power consumed but also, at constant amplitude, the devulcanization degree, and moved the gel–crosslink density points toward the main-chain-rupture curve (Table 2). Ultrasonically devulcanized EPDM roofing membrane (Table A1) gave 100% recycled revulcanizates with tensile strength similar to the virgin vulcanizate and elongation at break reduced from ~450% to 140–310%, depending on conditions [90].

The modeling study, on ground tire rubber and SBR, adopts cavitation as its working hypothesis for local bond breaking and states that the devulcanization mechanism and the conversion of mechanical ultrasonic energy to chemical energy are unclear [67], so cavitation remains a proposed mechanism.

The EPDM-direct ultrasonic evidence comes from continuous processing but from one laboratory in 2003 (Figure 6; Table A1 and Table A2). Other studies compare elastomers and process parameters [91,92]. On tire rubber, particle size significantly affected twin-screw ultrasonic outcomes [93], and the route has been optimized by response surface methodology [94]; both transfer as methods only.

Microwave treatment heats the bulk volumetrically and rapidly, but EPDM itself is scarcely responsive to the field and relies on carbon black for adequate heating [68,69]. EPDM-direct studies also cover compound development, blends with NR, chemical agents, paraffinic oil, and degradation kinetics [69,95,96,97,98].

Combined thermomechanical/microwave treatment of EPDM roofing waste, graded by crosslink density, sol fraction, Horikx plot, and thermogravimetric analysis (TGA) [38] (Table A1), is the most informative for selectivity. Compared with thermomechanical treatment alone, it was more efficient and more specific, its points lying around the crosslink scission curve. This placement supports crosslink over main-chain scission, but the authors’ reading of preferential action on disulfide bonds is a rank-level inference these metrics cannot confirm alone (Section 3.2). Treatment at 80 °C outperformed treatment at 110 °C, and the two-roll-mill study likewise rated its 80 °C devulcanizate best, with 100 °C least effective, and 120 °C close to 80 °C [35]; two EPDM studies with different energy delivery thus favor the milder setting, non-monotonically in one.

In the combined treatment, benzoyl peroxide is proposed to cleave homolytically to benzoyl and then phenyl radicals, which react with the weakest S–S bonds of the vulcanized EPDM and cut the crosslink; the pathway is taken from natural rubber work cited in [38], and no product analysis has demonstrated it. At 4 phr, the excess radicals react indiscriminately with S–S and C–C bonds, moving the samples toward the random scission curve, and the cage effect may re-crosslink what was cut. The source relates the stronger effect at 80 °C to peroxide surviving the milder thermomechanical step undecomposed for the microwave stage.

EPDM-direct microwave work remains at laboratory scale and is not graded on a common quality basis (Figure 6; Table A1 and Table 2). One EPDM-direct study names microwave and thermomechanical processing as the only routes productive enough for industrial use [35], though microwave devulcanization has not been industrialized [70]. The tire/NR/SBR/butyl microwave and high-energy literature [84,99,100,101,102,103,104,105,106,107,108,109,110] is cataloged at the review level [29].

### 4.4. Supercritical CO_2_, Solvent-Assisted, and Plasma Routes

Supercritical CO_2_, solvent-assisted, and plasma routes are limited by their substrate base. The first two replace or augment mechanical severity with a transport medium. Here scCO_2_ acts as a swelling carrier for devulcanization agents, in work that includes pilot-scale studies and a rapid-decompression route claimed in a patent [111,112,113]. Its evidence spans tread rubber, filled NR, isoprene, and butyl rubber, for which complete devulcanization is claimed [114], all of them non-EPDM [71,114,115,116,117,118,119,120].

Deep eutectic solvents (choline chloride–urea) with sonication were graded by Flory–Rehner crosslink density and Horikx selectivity [72] (Table 2). Terpinene has served in low-temperature mechanochemical reclamation of other elastomers [121]. For limonene, a bio-based solvent proposed to replace n-hexane in natural product extraction [122] and rated a low-toxicity food additive [123], the case rests on solvent properties and analogy alone. Hansen solubility-parameter matching has been demonstrated for SBR [124]. Autoxidation on air exposure also brings emission and handling consequences [122,125].

The proposed carrier and agent pathways come from tire rubber and NR. Below the critical pressure, the disulfide permeates tire rubber layer by layer, leaving the inner core almost untreated, whereas above it the agent reaches the interior, and devulcanization proceeds at the surface and inside simultaneously [71]. On cryo-ground tire rubber, the agent’s chemistry was inferred from Fourier-transform infrared spectroscopy (FTIR) and GC–MS as radical addition [49]. The benzenesulfide radical from thermal decomposition of diphenyl disulfide abstracts allylic hydrogen from the natural rubber chain or adds to its double bonds, and the polymer radical then undergoes crosslink scission but also main-chain degradation. The broader transport rationale draws on a model bilayer study [126].

The cluster’s primary substrates are tire rubber, NR, and other non-EPDM elastomers, and EPDM-direct performance is not established by these sources (Table A1). In situ high-pressure FTIR found EPDM to sorb CO_2_ at a level similar to the other elastomers measured, 15–20 wt% at 50 °C and 15 MPa, but on unfilled, uncrosslinked polymer, not on filled vulcanizate scrap [127]. This result removes one reason to expect transfer to fail but is no evidence that a tire-tuned window devulcanizes EPDM. Pretreatment is a further variable, since cryo-ground tire rubber morphology influences scCO_2_ outcomes [43,49].

An untested hypothesis follows from these sources: on EPDM, the scCO_2_ and solvent routes lack a monosulfide-active agent more than a carrier. Diphenyl disulfide is reported not to react with monosulfidic crosslinks in NR [116]. The EPDM vulcanizate of the NR–EPDM comparison began with 25% monosulfidic crosslinks, and 91% of the crosslinks surviving 275 °C were monosulfidic [6]. An amine [37] and low-concentration DBD [50] have been reported to break monosulfidic crosslinks in EPDM, the latter inferred from stress relaxation spectra of an efficiently cured network presumed mainly mono- and disulfidic. However, DBD is a diphenyl disulfide derivative, so the NR result need not carry to EPDM. Diphenyl disulfide also aided EPDM devulcanization only where heat alone already broke the network [5].

Dielectric-barrier-discharge plasma treatment of ground tire rubber, a non-thermal surface method distinct from the agent DBD, has been reported to assist network scission through reactive surface species [128]. The evidence is early stage, and EPDM performance has not been demonstrated (Table A1).

### 4.5. Comparison of Process Families

The families differ in how their sources propose the network is cut (bonds targeted in Table 3). Heat converts crosslinks to monosulfidic ones, and shear breaks them; ultrasound is proposed to act through cavitation. Disulfide-derived radicals combine with cut chains; an amine and, by stress relaxation inference, DBD also break monosulfidic crosslinks. Peroxide radicals attack S–S bonds, and a carrier delivers an agent to the interior.

The families also differ in demonstrated scale (Figure 6; Table A1). Each route is classed by the largest scale its source reports for it, and the classes are the review’s reading of the sources. Laboratory denotes batch processing in laboratory equipment, including a single extruded sample prepared only for comparison. Continuous or pilot denotes continuous processing studied as a route, with its process window, screw configuration, or throughput reported, or pilot-scale batch equipment, below a nameplate capacity of 100 kg/h. Semi-industrial denotes an industrial plant, a nameplate capacity of 100 kg/h or more, or an industrial batch charge. A route is regarded as replicated in continuous operation only when continuous operation on EPDM is reported by more than one laboratory.

By these criteria, two routes are replicated in continuous operation. Thermomechanical extrusion has been run continuously on EPDM in two laboratories [39,41]. The process of the first laboratory has also run on a 120 kg/h production line, which places the route in the semi-industrial class on energy data alone, since no devulcanizate quality was reported there [74].

Agent-assisted extrusion has been run continuously in three laboratories [7,8,51], the last on semi-industrial equipment, but with a different agent in each; no single agent chemistry has yet been run continuously on EPDM in a second laboratory. It also provides the only demonstrated compliance with the tested requirements of a rubber seal material specification (DIN EN 681-1, 60 Shore A category; not an automotive standard), met for tensile strength directly and for compression set and hardness only after blending with 25 wt% virgin rubber [8]. The same group later cited that specification as a target for liquid polymer activation without claiming compliance [83]. This specification evidence comes from a single group [8,9,50,83] and has not been independently replicated. A review also reports Toyota’s continuous reactive twin-screw extrusion of EPDM with reclaiming oil and agent (5–20 kg/h output capacity), whose reclaim could be used at up to 25% in virgin EPDM for low-demand automotive parts such as luggage-door weather strips [32].

Ultrasonic devulcanization of EPDM has run continuously, but only in one laboratory in 2003 [60,90], so it is continuous but not replicated. Microwave treatment has the broadest EPDM-direct base of the remaining routes, at least seven primary studies [38,68,69,95,96,97,98] all at laboratory scale, alongside a review-level overview [70]. A review of microwave rubber recycling notes that industrial application is still very limited after 40 years and calls for upscaling [129].

Supercritical CO_2_ has reached pilot-scale equipment only on scrap tire [111,118]. The scCO_2_, deep-eutectic-solvent and terpene routes [71,72,112,121], and plasma earlier still [128], remain unproven on EPDM.

## 5. Scale-up and Process Energy

The industrial relevance of the routes in Figure 6 turns on two coupled questions: whether a process can run continuously at meaningful throughput and at what energy cost. Table A2 lists both where reported. Continuous operation is documented most fully for extrusion, and the available energy accounting, sparse and drawn largely from analogous systems, points to process energy as a principal environmental lever and a cost factor. The 240 and 280 °C degradation thresholds of Section 4.1 refer to material temperatures, which differ from barrel set points (Table 2).

### 5.1. Batch and Continuous Operation

Five EPDM rows of Table A2 record continuous extrusion, and a sixth records a continuous ultrasonic reactor [90]. Thermomechanical co-rotating twin-screw devulcanization of post-industrial EPDM has been run at 3.5 kg/h across systematic screw profile and thermal profile variations [39]. An amine route was taken into a co-rotating twin-screw extruder at 3–7 kg/h and a 250–300 °C set temperature. There, a kinetic model derived from batch data predicted the decrosslinking degree with a standard error of 4% inside the batch temperature range but not on extrapolation beyond it [7]. A DBD route optimized in a 40 g batch was translated to a continuous process at 270 g/h, far below industrial throughput, with flow rate, screw speed, and temperature re-tuned [8]. At semi-industrial scale, a twin-screw extruder processed waste automotive EPDM powder continuously with an MBTS agent [51]. The fifth row is the 120 kg/h production line of the only EPDM life cycle study [74]. With the twin-screw arm of a batch-versus-continuous comparison [41] (Table A1), these rows form the continuous EPDM-direct record behind the classes of Figure 6.

**Figure 7:**
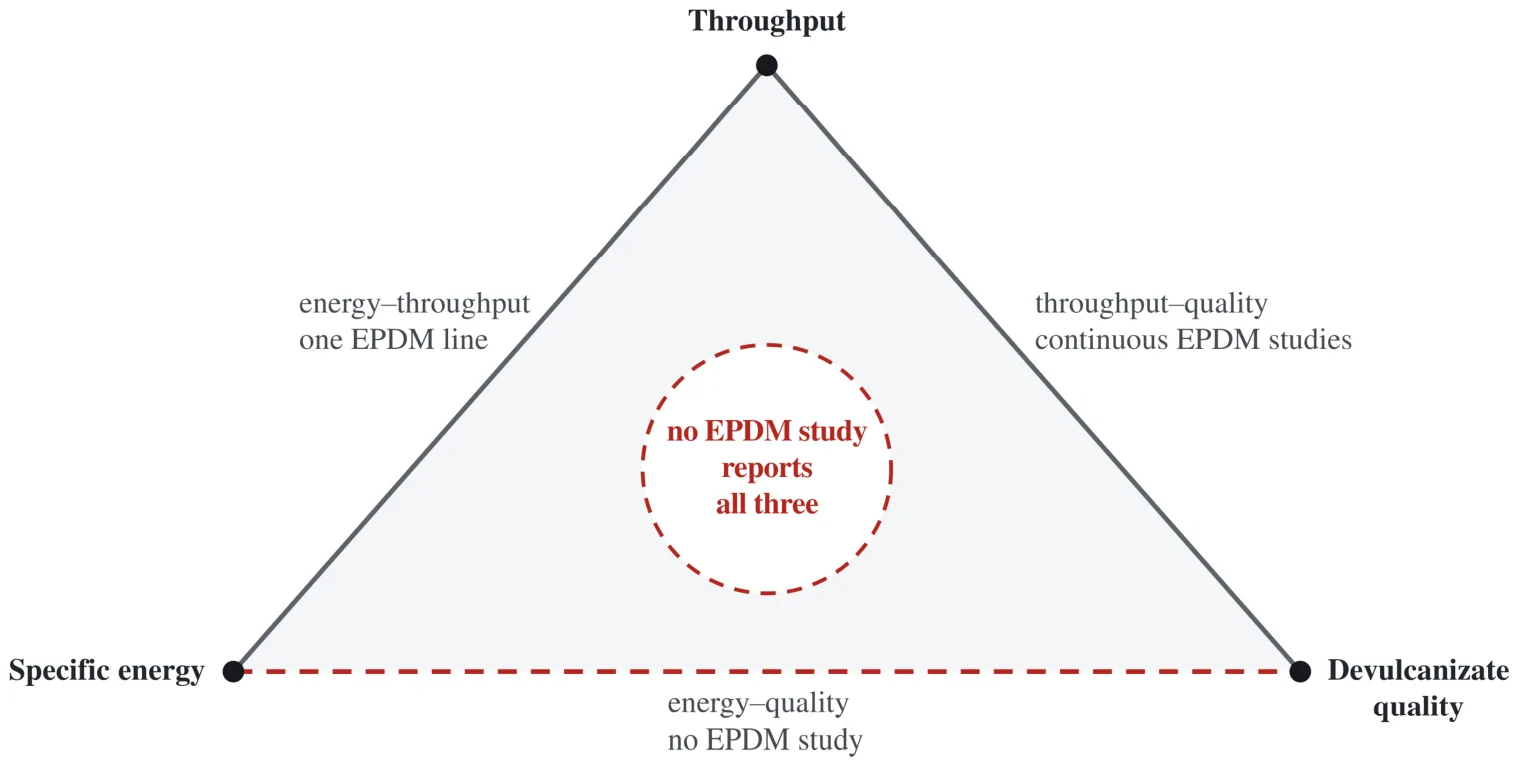
Pairwise reporting of throughput, specific energy, and devulcanizate quality (the metric set of Table 2) in EPDM devulcanization studies (schematic). Solid edges, pairs reported in EPDM studies: throughput–quality (continuous demonstrations) and energy–throughput (one peer-reviewed EPDM line with a measured electricity inventory). Dashed edge, specific energy–quality, paired in no EPDM study; transfer-level microwave studies pair specific energy with crosslink density decrease [99,103]. Dashed circle, no EPDM study reporting all three.

No row reports throughput, per-kilogram energy, and quality together (Figure 7). The production line reports energy but no devulcanizate quality, and the semi-industrial extrusion reports quality but neither run throughput nor energy (Table A2). Scaling to continuous operation may also trade away some selectivity. In the only direct batch-versus-continuous comparison, the batch processes cleaved sulfur crosslinks somewhat more effectively than extrusion (75% against 72.5%, with slightly more chain degradation in extrusion) for one profile-grade compound [41]. Closing that gap is an open process-engineering problem. A complementary template comes from tire rubber, where a batch devulcanization of ground passenger car tire rubber was carried over explicitly to continuous operation [130]. The documented batch-to-continuous methodology transfers to EPDM, but its substrate-specific numbers do not.

### 5.2. Specific Mechanical Energy and Process Energy Data

The specific mechanical energy (SME), drive-motor power divided by throughput, has been used to follow the progress of ground tire rubber reclamation [131]. However, it counts drive-motor power only and can move opposite to total extruder consumption, so it is not a cost proxy. For twin-screw thermomechanical treatment of ground tire rubber, specific mechanical energy has been quantified per parameter set at 0.006–0.053 kWh/kg, with total extruder energy consumption at 0.157–0.178 kWh/kg [131]. The barrel was fixed at 200 °C and the throughput at 2 kg/h, and the values varied with screw speed and oil loading. The absolute numbers transfer to EPDM only as an order-of-magnitude and methodological guide. Nothing in the reviewed data shows that an energy-minimizing and a selectivity-maximizing set point coincide, and no study optimizes both jointly; scale-up is a multi-objective problem (throughput, energy, selectivity).

The energy column of Table A2 is nearly empty for EPDM. The peer-reviewed EPDM studies report process conditions and quality outcomes but rarely a per-kilogram energy figure. Only the life cycle study reports throughput and per-kilogram energy together. Its industrial line produced 120 kg/h, and its measured electricity inventory of roughly 0.9 kWh/kg is dominated by the extruder motor and barrel heaters [74]. Supplier and trade figures lack a citable record. Network breakdown quality comes from the same group’s laboratory-scale paper, on washing machine seals in a 20 mm extruder [39], so the throughput–energy–quality dataset a process design requires must be assembled from several papers (Figure 7).

### 5.3. Process Energy and Life Cycle Assessment

Only one dedicated process life cycle assessment (LCA) was found for EPDM, so the environmental case rests on a single quantitative study. It covers automotive gasket waste on the production line of Table A2, with 120 kg of devulcanized EPDM produced in 1 h as the functional unit and electricity from the Italian national grid [74]. Within that boundary, the main contribution to the environmental impacts is energy consumption, above all for heating and rotating the extruder screws [74]. The recycled route was lower in every impact category in a cut-off comparison per 1 kg of EPDM compound and under system expansion over two product life cycles, with incineration credited for recovered heat and electricity; Monte Carlo probabilities of a lower impact under the latter exceeded 94% except in freshwater eutrophication (72%) [74]. The result is conditional on a 30 wt% blend assumed to match virgin properties, on the Italian grid mix, and on the exclusion of collection, sorting, and transport, and it has not been replicated. The study reports no devulcanizate quality, so its energy figure cannot be tied to selectivity (Table A2).

Tire recycling LCAs, which concern a transfer substrate, show which method choices decide such comparisons. Two such studies define the functional unit per tonne of end-of-life tires and draw the boundary from collection and transport through pretreatment and treatment to the substitutes for the recovered products [132,133]. Multifunctionality is resolved by system expansion with avoided burden credits, whose weak point is that assumptions about the substituted process can make LCAs of the same topic diverge [132]. Credits often contributed most to the results. Credits for energy recovery shrink as the share of renewables in the energy mix grows, and recovered carbon black replaces virgin grades only to a certain degree [132]. Crumb rubber treated as burden-free waste and replacing 50 vol% of the river sand in a cement composite cut global warming potential by 24.06%, while the composite’s physical–mechanical properties fell by 40% [133]. A review of tire recycling LCAs finds that many studies are gate-to-gate with a single functional unit and lists process energy consumption and the carbon intensity of electricity among sensitivity inputs [134].

From these precedents and the metrics of Table 2, a minimum reporting set is proposed for an EPDM devulcanization LCA, ordered by the four phases of ISO 14040/14044 [135,136] that the EPDM study followed [74] (Table 4). Its central choice is a quality-graded functional unit, 1 kg of devulcanizate of a declared grade (Δ*ν*, sol fraction, Horikx position, and Mooney viscosity) or 1 kg of compound meeting a stated specification. A unit per tonne of waste treated cannot distinguish a selective devulcanizate from a degraded one, and the EPDM study already compared routes per kilogram of compound, without a quality grade. The boundary runs from collected scrap, including grinding (Table 1), to the devulcanizate for process comparison, and to a compound meeting a stated specification whenever substitution credits are claimed; it is a minimum, narrower than the cradle-to-grave boundary recommended for tire recycling LCAs [134].

The inventory itemizes motor and heater electricity separately, together with agents, oils, virgin rubber in blends, and transport. The credit for displaced virgin EPDM scales with blend ratio and quality, and the credit for averted disposal is reported separately. Sensitivity runs cover grid mix, blend ratio, transport, and the coupling of energy to quality. Reporting throughput, per-kilogram energy, and quality together would also fill the empty center of Figure 7. However, a product-level claim that devulcanized EPDM is environmentally preferable requires the recycled-content compound’s full data package (LCA/product carbon footprint, recycled-content accounting, chain of custody), which process-side accounting cannot settle.

## 6. Conclusions and Future Perspectives

Sulfur-cured EPDM can be devulcanized with predominantly crosslink scission, although the EPDM-direct evidence is narrower, and the scale it reaches is smaller than the wider rubber recycling literature suggests. In the only direct comparison, ENB-EPDM was much less reactive toward disulfides than NR [6], so for disulfide-type routes, NR and tire results may overstate the ease of devulcanizing EPDM. Swelling crosslink density, sol fraction, Horikx selectivity analysis, and a rheological proxy together grade devulcanizate quality on the axis relevant to recycling, selective crosslink scission against main-chain degradation. Grade classes are still lacking, so studies should report all four metrics together and state the filler context. Two EPDM studies rated an 80 °C devulcanizate best against 100–120 °C, non-monotonically in one [35,38]; in twin-screw extrusion, selectivity depended on thermal profile and screw speed jointly. The usable process window, bounded by under-devulcanization and by main-chain scission and re-crosslinking, therefore has to be located by measurement on the compound and equipment in use. Only thermomechanical and agent-assisted extrusion have run continuously on EPDM in more than one laboratory, the latter with a different agent in each. At 270 g/h, one group’s agent-assisted extrusion has also met the tensile, compression set and hardness requirements of a non-automotive rubber seal specification, the last two only after blending with 25 wt% virgin rubber, a result not independently replicated [8]. Ultrasonic processing rests on one laboratory and microwave treatment on laboratory-scale studies without common metric grading; the scCO_2_, solvent-assisted and plasma routes remain unproven on EPDM. The one EPDM process LCA (functional unit, 120 kg of devulcanized EPDM in 1 h; Italian grid) found energy consumption the main contributor to impacts [74].

Future work should (i) define standard grade classes and reference states; (ii) obtain EPDM-direct performance data for the routes advanced on environmental grounds (scCO_2_, deep eutectic solvents, and terpenes), where a monosulfide-active agent may matter more than the carrier; (iii) calibrate an in-line proxy to selectivity for continuous lines; (iv) report throughput, specific energy, and quality together for one EPDM line, with an LCA following the minimum reporting set of Table 4; and (v) develop a feedstock-routing protocol for post-consumer EPDM. Beyond these five gaps, EPDM product analyses of agents other than diphenyl disulfide would turn proposed scission pathways into demonstrated ones. Closing these gaps, and replicating one agent chemistry in a second laboratory, would make agent-assisted continuous extrusion of sorted, sulfur-cured EPDM scrap a specifiable feedstock route; whether its devulcanizate meets the property and data package requirements of automotive parts is then a compounding and qualification question.

## Figures and Tables

**Table 3:** Process classes for breaking down sulfur-cured rubber networks. Classes and control ratings are this review’s synthesis of the cited sources. EPDM evidence labels: EPDM-direct, transfer only (tire, NR, or SBR), none found in the sources consulted, and not reviewed (pyrolysis, outside the review’s scope). PB = polybutadiene.

Process Class (Product)	Bonds Targeted	Control over Selectivity	Main Advantage	Main Limitation	EPDM Evidence	Refs.
Thermomechanical, heat and shear (devulcanizate for revulcanization)	S–S, C–S; many C–C bonds also break under heat alone	Moderate; thermal profile, screw speed and screw design jointly set severity and selectivity	Continuous, easy to industrialize, no agents or solvents	Outside the window, rubber too crosslinked or too devulcanized for revulcanization	EPDM-direct	[35,39,41]
Agent-assisted mechanochemical, disulfide or amine (devulcanizate)	S–S, C–S; amine also monosulfidic crosslinks	Moderate; agent type, concentration, and temperature	Run on a semi-industrial extruder (nameplate 100–250 kg/h)	Low disulfide efficiency on EPDM; odor; chemical toxicity; mechanisms not fully understood	EPDM-direct	[7,8,18,37,50,51]
Ultrasonic and microwave (devulcanizate)	S–S, C–S; main chains also, more so with carbon black (ultrasonic) or excess peroxide radicals (microwave)	Moderate; ultrasonic amplitude; peroxide dose with microwaves	Ultrasonic continuous without chemicals; microwave very fast	Specialized equipment; EPDM absorbs microwaves poorly without carbon black; microwave not industrialized; ultrasonic mechanism unclear	EPDM-direct	[38,60,67,68,69,70]
scCO_2_ and other solvent-assisted (devulcanizate)	S–S, C–S through a carried agent; main chains also cut	Not rated for EPDM; pressure sets agent penetration into the particle	Agent reacts inside the particle above the critical pressure; non-toxic, biodegradable deep eutectic solvent	No EPDM-direct performance data	Transfer only	[71,72]
Reclaiming, traditional (reclaim used as rubber diluent, extender or processing aid)	Crosslinks and main chain	Low; main-chain scission occurs along with crosslink scission	Long-established technology for NR	NR reclaiming chemistry not transposable to EPDM	EPDM-direct, negative	[6,21,37]
Biological, bacteria, or fungi (surface-devulcanized particles)	S–S, C–S at the particle surface	High in principle, limited in time and to the surface	Mild, low-temperature conditions; environmentally friendly	Low devulcanization ratio, contamination risk, surface-limited, very slow (weeks); not industrial	None found (ground tire rubber, NR, and latex reported)	[18,19,66]
Metathesis depolymerization (soluble polymers or telechelic oligomers)	Backbone C=C (catalyst attacks the double bond)	High over-product molecular weight (catalyst and co-reactant olefin); not crosslink-selective	Room temperature, about 2–3 h, minimal side degradation	Requires backbone C=C; reported for polybutadiene, natural and isoprene rubber, SBR, and nitrile rubber in the reviews consulted, soluble product only for polybutadiene among vulcanized PB and SBR	None found; EPDM’s residual C=C is pendant to a saturated backbone (Figure 2a)	[3,18,19,20,66]
Pyrolysis (oil, gas, and carbon-black char)	All organic bonds (thermal decomposition without O_2_)	None; decomposition is irreversible	Energy, fuel, or chemicals from waste	Low product quality; energy recovery, not recycling, under Directive 2008/98/EC	Not reviewed	[15,20]

**Table 4:** Minimum reporting set proposed in this review for life cycle assessment (LCA) of EPDM devulcanization, derived from the one EPDM process LCA and from tire recycling LCAs (transfer) and ordered by the phases of ISO 14040/14044, with reporting last. A dash in Refs. marks a row without a source precedent.

ISO 14040/14044 Phase: Element	Proposed Choice for EPDM Devulcanization	Rationale (Link to This Review)	Refs.
Goal and scope: functional unit	1 kg of devulcanizate of a declared grade (Δ*ν*, sol fraction, Horikx position, Mooney viscosity), or 1 kg of compound meeting a stated specification	Quality is a position on the scission trade-off (Table 2); units per tonne of waste or per hour of output do not carry it; the EPDM LCA compared routes per kilogram of compound, without a quality grade	[74,132,133]
Goal and scope: system boundary	Collected scrap, including grinding, to devulcanizate for process comparison; to a compound meeting a stated specification whenever substitution credits are claimed	Comminution history conditions quality (Table 1); one tire LCA set crumb-preparation energy to zero, and the EPDM LCA excluded collection, sorting, and transport; a cradle-to-grave boundary is recommended for tire LCAs	[74,133,134]
Inventory: per kg	Electricity split into drive-motor and barrel heaters; devulcanization agents; process oils; virgin rubber in blends; transport	The motor and heaters dominate the one EPDM inventory, and SME counts motor power only; chemical additives can carry environmental risks of their own	[74,131,137]
Inventory: multifunctionality and credits	System expansion; credit for displaced virgin EPDM scaled by blend ratio and quality (substitution ratio); credit for averted disposal reported separately	Credits can dominate results and depend on substitution assumptions; the EPDM result rests on a 30 wt% blend assumed equivalent to virgin compound	[74,132]
Impact assessment: method	Declared midpoint method, e.g., Environmental Footprint 3.0; results per category; toxicity categories with their uncertainty	Freshwater ecotoxicity was assessed in the EPDM LCA, whereas toxicity was excluded from a tire LCA because of its uncertainty	[74,132]
Interpretation: sensitivity	Grid mix, blend ratio, transport distance, energy–quality coupling (energy at two or more quality grades)	Energy is the main contributor, so grid mix sets its weight; renewable shares change credits; no EPDM study ties per-kilogram energy to quality (Figure 7)	[74,132,134]
Reporting	Throughput, per-kilogram energy, and quality grade for one line, with the inventory	The center of Figure 7 is empty; energy figures at different quality grades are not comparable	—

## Data Availability

No new data were created in this review. The comparison tables supporting the review are included in the article (Appendix A and Appendix B); the published articles and third-party figure panels analyzed remain available from their original publishers under the applicable license terms.

## Abbreviations

The following abbreviations are used in this manuscript:

BPO	benzoyl peroxide
BTEX	benzene, toluene, ethylbenzene, xylenes
CB	carbon black
ChCl	choline chloride
cTSE	co-rotating twin-screw extruder
DBD	dibenzamido-diphenyl-disulfide
DCPD	dicyclopentadiene
DES	deep eutectic solvent
DPDS	diphenyl disulfide
EM	emerging/solvent-assisted (Table A1 route code)
ENB	5-ethylidene-2-norbornene
EPDM	ethylene–propylene–diene monomer rubber
EU	European Union
EV	efficient vulcanization
FTIR	Fourier-transform infrared spectroscopy
GC–MS	gas chromatography–mass spectrometry
GPC	gel permeation chromatography
GTR	ground tire rubber
HD	1,4-hexadiene
HDA	hexadecylamine
HZ	heating zone
LCA	Life cycle assessment
MBTS	2-mercaptobenzothiazole disulfide
MC	mechanochemical (Table A1 route code)
MDR	moving die rheometer
MW	microwave (Table A1 route code)
NR	natural rubber
PB	polybutadiene
phr	parts per hundred rubber
RWP	rubber waste particles
SBR	styrene–butadiene rubber
scCO_2_	supercritical carbon dioxide
SME	specific mechanical energy
SSA	specific surface area
TGA	thermogravimetric analysis
TM	thermomechanical (Table A1 route code)
TMTD	tetramethylthiuram disulfide
TSE	twin-screw extruder
TSSR	temperature scanning stress relaxation
US	ultrasonic (Table A1 route code)
VOC	volatile organic compound
VR	virgin rubber

## Appendix A

**Table A1:** EPDM devulcanization by route: substrate, reported conditions, network breakdown outcomes and maturity class. Route codes: TM, thermomechanical; MC, mechanochemical; US, ultrasonic; MW, microwave; EM, emerging/solvent-assisted; plasma, uncoded. Substrate: EPDM, demonstrated on EPDM; EPDM(t), EPDM crumb of tire origin; tr, tire or ground tire rubber (GTR) transfer; NR, natural rubber transfer; review, review-level source; single group or single laboratory, EPDM-direct result not independently reproduced. Class, largest scale the cited source reports for the route (criteria in Section 4.5), as plotted in Figure 6. n/a, not applicable; ↑, increase; ↓, decrease. Values as reported (*ν* in mol/m^3^); grading protocols differ between studies (Table 2).

Route	Substrate	Reported Conditions	Δ*ν*/% Devulcanization	Sol, Selectivity, or Properties	Scale/Throughput	Class	Refs.
Two-roll mill (TM)	EPDM	80 °C, 1.33 friction; 100 °C least effective	Δ*ν* ≈ 53%	Sol 5.4 wt% (others as low as 3–5 wt%); selective (Horikx)	Lab batch	Laboratory	[35]
Batch mill + internal mixer (TM)	EPDM	Batch route vs. TSE on one profile grade	Δ*ν* ≈ 75%	Little degradation (Horikx)	Lab batch	Laboratory	[41]
Co-rotating TSE (TM)	EPDM	Thermal profile, rpm and screw profile varied; in a second laboratory, TSE compared with the batch route	↑ with *T* and rpm; TSE 72.5% vs. batch 75%	Sol/random scission fraction process-dependent	Continuous; 3.5 kg/h (first study)	Continuous or pilot	[39,41]
Production line TSE (TM)	EPDM	Automotive gasket waste, 58 mm co-rotating TSE, no agents	Not reported (LCA study)	Not reported	Industrial line, 120 kg/h	Semi-industrial (energy data only)	[74]
Internal mixer + amine aid (MC)	EPDM	Conventionally cured EPDM, hexadecylamine, 75 rpm, 7.5 min, 225–275 °C	Δ*ν* 46–51% (*ν* 239 → 116–128 mol/m^3^), decrease mainly thermal; breakdown to measurable Mooney needed the amine	Poly/disulfidic broken → some monosulfidic; amine also breaks monosulfidic	Lab batch	Laboratory	[37]
TSE, agent (TM/MC)	EPDM(t)	Twin-screw extrusion of used-tire EPDM crumb	Degree of regeneration 80–87%	Shear-intensity dominated	Continuous co-rotating TSE (25 mm)	Continuous or pilot	[47]
Reactive TSE + oil/agent (TM/MC)	EPDM; review	Rubber/oil/agent 20:6:1, pre-swollen ≥ 24 h; 300 °C, 10 min	Gel fraction 45%; effective network chain density 1/20 of virgin	Linear chains (molecular-weight distribution close to raw rubber) ≈ 1:1 with a loose network; crosslinks thought selectively, not completely, broken	Continuous TSE, output capacity 5–20 kg/h	Continuous or pilot	[32]
TSE + DBD (MC)	EPDM; single group	2 wt% DBD, 120 → 80 °C, 50 rpm, 4.5 g/min	Not stated as Δ*ν*	Tensile met DIN EN 681-1 (non-automotive seal spec); compression set and hardness only after 25 wt% VR blending	40 g batch → 270 g/h	Continuous or pilot	[8]
Mixer + DBD + liquid polymer (MC)	EPDM; single group	0–3.9 wt% DBD, 120–200 °C; liquid polymer 0–15 wt%	Degree of devulcanization (storage modulus basis) up to 64%	Tensile strength and strain at break rise with network breakdown, except a strain-at-break drop between 53% and 64% devulcanization (residual DBD)	Lab; one extruded sample (3 g/min) for comparison	Laboratory	[83]
Heated press + DBD (MC)	EPDM; single group	EV-cured, carbon-black-filled EPDM waste powder; 0.4–13.8 wt% DBD, 180 °C, 15 min (press); 150–200 °C at 3.9 wt% (rheometer)	*ν* (TSSR) 201 → 52 mol/m^3^ from 0 to 13.8 wt% DBD at 180 °C	Monosulfidic crosslinks cleaved at low DBD, polysulfidic needing more (TSSR relaxation spectra; network composition presumed)	Lab batch	Laboratory	[50]
Thermal ± DPDS or HDA (MC)	EPDM	Unfilled ENB-, HD- and DCPD-EPDM, conventional sulfur cure (ENB- and HD-EPDM also peroxide cured); closed mold, 200 or 265 °C, 2 h	Diene-dependent; DCPD-EPDM further crosslinks; sulfur HD-EPDM complete with ≥2 × 10^−4^ mol/cm^3^ DPDS at 265 °C	Sulfur ENB-EPDM mainly crosslink scission; sulfur HD-EPDM crosslink and main-chain scission; peroxide ENB-EPDM mainly random, peroxide HD-EPDM crosslink scission (Horikx)	Lab batch	Laboratory	[5]
TSE + amine (MC)	EPDM	EV-cured, 110 phr CB; hexadecylamine; 250–300 °C set, 50–200 rpm	Decrosslinking degree predicted by a batch-derived kinetic model (standard error 4%) within 225–285 °C	One devulcanizate (300 °C set, 50 rpm, 3.5 kg/h): GPC molecular weight 23,000 vs. 58,000 g/mol virgin	Continuous, 3–7 kg/h	Continuous or pilot	[7]
Semi-industrial TSE + MBTS (MC)	EPDM; single group	MBTS 6 phr, 220 °C, main screw 180/220 rpm	Δ*ν* ≈ 92% (*ν* 186 → 14.5–15.2 mol/m^3^); devulcanization 91.9–92.2%	Sol 13.1–17.6 wt%; blend thresholds vs. automotive reference compound reported	Semi-industrial TSE (nameplate 100–250 kg/h)	Semi-industrial	[51]
Steam autoclave + MBTS (MC)	EPDM; single group	Oil-soaked automotive EPDM powder, 250 kg charge; 165 °C/6 bar or 175 °C/8 bar steam, 8 h	Devulcanization 74–95% at 175 °C/8 bar (76% with TMTD, 94% with MBTS, equal oil ratio); *ν* 186 → 10–48 mol/m^3^	MBTS more effective than TMTD; radical scheme proposed	Industrial batch	Semi-industrial	[40]
Ultrasonic reactor (US)	EPDM; single laboratory	Continuous grooved-barrel reactor; amplitude varied	Gel/*ν* ↓ with amplitude	CB lowers US power	Continuous, ≤2.52 g/s (roofing membrane)	Continuous or pilot	[60,90]
Microwave (MW)	EPDM; review; tr	Combined TM/MW; BPO 0/2/4 phr, optimum 2 phr (4 phr less effective); 80 and 110 °C	Greater Δ*ν* than TM alone	Near crosslink scission curve (Horikx); disulfide selectivity and main-chain sparing inferred by the authors	Lab batch	Laboratory	[38,68,69,70,95,96,97,98,101,102]
scCO_2_ (EM)	tr; NR	Supercritical CO_2_ carrier/medium	n/a	Transfer only; CO_2_ sorption of neat EPDM comparable to other elastomers (unfilled, uncrosslinked)	Pilot-scale extraction unit, 1.5 kg crumb (scrap tire); otherwise, lab static autoclave	Continuous or pilot	[71,111,112,115,118,127]
DES/terpene (EM)	tr (NR sheets only for the terpinene swelling test)	ChCl–urea + sonication; terpinene	Flory–Rehner/Horikx reported	Transfer only	Lab batch	Laboratory	[72,121]
Plasma (early)	tr (GTR)	Dielectric-barrier discharge, 18 kV, 30 min, atmospheric	Δ*ν* ≈ 62% (*ν* 210 → 80 mol/m^3^)	S–S/C–S cleavage claimed; surface-acting; early stage	Lab batch	Laboratory	[128]
Reference TSE (TM)	NR	TSE termed semi-industrial by the source, 80–220 °C barrel	≈90% reclaiming	Best selectivity at low input *T* (self-heating)	5 kg/h run; machine max 30 kg/h	Continuous or pilot (context only)	[62]

## Appendix B

**Table A2:** Scale-up and process energy data for devulcanization: throughput, energy, and their coupling to quality. Route codes and substrate labels as in Table A1; none, no energy–quality pairing in that study.

Route (Code; Substrate)	Scale/Throughput	Specific/Total Energy	Quality–Energy Link	Note	Refs.
TSE + DBD (MC; EPDM)	40 g batch → 270 g/h continuous TSE	Not reported	None (no energy figure)	20 mm laboratory compounder; flow rate, screw speed, and temperature re-tuned	[8]
TSE + amine (MC; EPDM)	Co-rotating TSE, 3–7 kg/h	Not reported	None; decrosslinking degree modeled against conditions	Batch-derived kinetic model	[7]
Semi-industrial TSE + MBTS (MC; EPDM)	Semi-industrial TSE (nameplate 100–250 kg/h; run rate not reported)	Not reported	None (no energy figure)	Automotive EPDM powder feedstock	[51]
Co-rotating TSE, laboratory (TM; EPDM)	Continuous TSE, 3.5 kg/h (all tests); screw and thermal profiles varied	Not reported	None; quality mapped to conditions	%sol rises with thermal profile (to ~31%); %random shows no global severity trend	[39]
Production line TSE (TM; EPDM)	Industrial cTSE, 120 kg/h	≈0.9 kWh/kg, itemized measured electricity	None; quality not reported in the same study	Same group and process as the row above; 58 mm vs. 20 mm extruder	[74]
Ultrasonic reactor (US; EPDM)	Continuous reactor, ≤2.52 g/s	Ultrasonic power per horn, not per kilogram	Gel fraction, *ν*, and tensile reported	Specialized reactor	[90]
Twin-screw thermomechanical (TM; tr)	2 kg/h	SME 0.006–0.053 kWh/kg; total extruder energy 0.157–0.178 kWh/kg	None (surface chemistry only); energy varies with screw speed and oil loading	Transfers to EPDM as order of magnitude and method only	[131]
Reference TSE (TM; NR)	TSE termed semi-industrial by the source	“low energy consumption” (qualitative)	≈90% reclaim at every barrel *T*; selectivity best at low input *T*	Self-heating decouples material *T* from barrel *T*	[62]
